# A mixed-valence [Co^II^_4_Co^III^_2_] cluster with defect disk-shaped topology

**DOI:** 10.1107/S2053229622005885

**Published:** 2022-08-26

**Authors:** Hua Yang, Yu-Pei Fu, Yuan Huang, Xiao-Li Chen, Dan Qiao, Hua-Li Cui

**Affiliations:** a School of Chemistry and Chemical Engineering, Shaanxi Key Laboratory of Chemical Reaction Engineering, Laboratory of New Energy and New Function Materials, Yan’an University, Yan’an 716000, People’s Republic of China; b College of Chemistry, Chemical Engineering and Materials Science, Soochow University, Suzhou 215123, People’s Republic of China; University of Sydney, Australia

**Keywords:** hexa­nuclear com­plex, cobalt, metal cluster, defect disk-shaped topology, crystal structure, magnetic properties

## Abstract

The employment of the new Schiff base ligand 2-[(4-chloro-2-hy­droxy­benzyl­idene­amino)­meth­yl]phenol (H_2_ *L*) bearing O_2_N donors for the preparation of a novel Co_6_ cluster is reported. The hexa­nuclear cobalt com­plex, namely, di-μ_2_-acetato­tetra­kis­{μ_2_-2-[(4-chloro-2-oxido­benzyl­idene­amino)­meth­yl]phenolato}tetra-μ_3_-methano­lato-tetra­cobalt(II)dicobalt(III), [Co^II^_4_Co^III^_2_(C_14_H_10_ClNO_2_)_4_(CH_3_COO)_2_(CH_3_O)_4_], was obtained using Co(CH_3_COO)_2_·4H_2_O and H_2_ *L* as starting materials in MeOH under solvothermal conditions. The six metal ions are linked together by the μ_3_-O atoms of four deprotonated MeOH mol­ecules, two CH_3_COO^−^ units and six phenolate O atoms of four *L* ^2−^ ligands to form a defect disk-shaped topology. DC magnetic susceptibility investigations revealed the existence of anti­ferromagnetic inter­actions in the Co_6_ cluster.

## Abstract 2

A mixed-valence Co_6_ cluster was synthesized and characterized. It exhibits a defect disk-shaped architecture. DC magnetic property studies in the 2.0–300 K range revealed anti­ferromagnetic inter­actions between the Co^II^ ions.

## Introduction

Polynuclear coordination com­pounds of 3*d* transition metals have attracted continued attention for several decades due to their structural novelty, inter­esting catalytic (Dastidar & Chattopadhyay, 2022 ▸; Shul’pin & Shul’pina, 2021 ▸; Nesterov & Nesterova, 2018 ▸; Jing *et al.*, 2020 ▸) and biological properties (Hazari *et al.*, 2017 ▸; Amtul *et al.*, 2002 ▸; Azizian *et al.*, 2012 ▸; Tanaka *et al.*, 2003 ▸), and their potential as single-mol­ecule magnets (SMMs) (Radu *et al.*, 2017 ▸; Pattacini *et al.*, 2011 ▸). Among numerous polynuclear 3*d* com­plexes, cobalt clusters have received particular inter­est because of their pleasing topological aesthetics (Brechin *et al.*, 1997 ▸; Cao *et al.*, 2013 ▸), their relevance to di­oxy­gen reduction (Monte-Pérez *et al.*, 2017 ▸) and their fascinating magnetic properties (Liu *et al.*, 2020 ▸; Sarto *et al.*, 2018 ▸; Li *et al.*, 2020 ▸; Ma *et al.*, 2012 ▸).

Several synthetic methodologies towards polynuclear cobalt clusters were established and one of the most efficient approaches involves the employment of hy­droxy-containing Schiff base ligands. Schiff base ligands are easy to synthesize and their steric properties can be tuned by varying the size of the amine or carbonyl substituents (Qin *et al.*, 2017 ▸, 2018 ▸; Ge *et al.*, 2018 ▸; Li *et al.*, 2021 ▸). More importantly, the hy­droxy moieties of the Schiff base ligands can combine many metal ions with μ-O bridges, resulting in the formation of large polynuclear clusters.

In the present work, we utilized the hy­droxy-containing Schiff base 2-[(4-chloro-2-hy­droxy­benzyl­idene­amino)­meth­yl]phenol (H_2_ *L*) (Huang *et al.*, 2019 ▸) as a ligand to assemble a polynuclear cobalt cluster. The hexa­nuclear cobalt com­pound [Co_4_^II^Co_2_^III^(*L*)_4_(CH_3_COO)_2_(MeO)_4_] (**1**) was obtained suc­cessfully and we report its structural diversity and discuss its magnetic properties.

## Experimental

### Materials and physical measurements

All chemicals were of reagent grade, purchased from commercial suppliers and used without further purification. All manipulations were conducted under aerobic and solvothermal conditions. H_2_ *L* was synthesized following the liter­a­ture procedure of Huang *et al.* (2019 ▸). Elemental analyses for C, H and N were performed with a Carlo-Erba EA1110 CHNO-S analyser. The FT–IR spectrum was determined on a Nicolet MagNa-IR 500 spectrometer using KBr pellets in the range 400–4000 cm^−1^. DC magnetic susceptibilities were measured in the temperature range 2–300 K in a field of 1000 Oe using a Quantum Design MPMS-7 SQUID magnetometer.

### Synthesis and crystallization

To a Pyrex tube (10 ml) was added a mixture of H_2_ *L* (0.0291 g, 0.1 mmol), Co(CH_3_COO)_2_·4H_2_O (0.0249 g, 0.1 mmol), Et_3_N (0.0202 g, 0.2 mmol) and MeOH (1.5 ml). The tube was sealed and heated at 80 °C for 48 h under autogenous pressure. It was then cooled to room temperature and dark-red needle-like crystals were obtained. The crystals were collected, washed with MeOH (2 ml) and dried in air (yield: 0.020 g; 48% based on cobalt). Analysis calculated (%) for C_64_H_58_Cl_4_Co_6_N_4_O_16_: C 47.03, H 3.58, N 3.43; found (%): C 46.18, H 4.048, N 3.280. Selected IR data for **1** (cm^−1^): 1637 (*s*), 1590 (*s*), 1523 (*s*), 1450 (*m*), 1419 (*m*), 1286 (*w*), 1248 (*s*), 1185 (*s*), 1089 (*m*), 1021 (*m*), 933 (*s*), 874 (*m*), 852 (*w*), 755 (*s*).

### Structure determination

Crystal data, data collection and structure refinement details are summarized in Table 1 ▸. The crystal structure contained disordered solvent that could not be satisfactorily refined. The SQUEEZE (Spek, 2015 ▸) routine of *PLATON* (Spek, 2020 ▸) was used in the treatment of the crystallographic data. All H atoms were placed in geometrically idealized positions, with C—H = 0.95–0.99 Å. The H atoms of the CH_2_, aromatic and amide groups were constrained to ride on their parent atoms, with *U*_iso_(H) = 1.2*U*_eq_(C). The H atoms of CH_3_ groups were refined as rotating groups, with *U*_iso_(H) = 1.5*U*_eq_(C).

## Results and discussion

### Synthesis of com­plex 1 and IR spectral analysis

The reaction of H_2_ *L* and Co(CH_3_COO)_2_·4H_2_O in MeOH in the presence of NEt_3_ under solvothermal conditions led to the isolation of **1** in moderate yield. Co(CH_3_COO)_2_·4H_2_O is a good starting material because it not only serves as a convenient metal source, but also provides CH_3_COO^−^ bridging ligands. In the solid state, com­plex **1** is stable in air and its elemental analysis is consistent with the given mol­ecular formula.

The vibrational bands in the IR spectrum agree well with the formulation of com­plex **1** (see Fig. S1 in the supporting information). The signals of the carb­oxy­l ν_as_(CO_2_) and ν_s_(CO_2_) vibrations were found in the 1637–1419 cm^−1^ range. The vibrations of the C=N bond appear at 1450 cm^−1^. Several bands in the 1286–1185 cm^−1^ range were assigned to the vibrations of the aromatic rings. The sharp signals in the 979–766 cm^−1^ range were ascribed to the vibrations of C—H bonds.

### Structure description of 1

**Figure 1:**
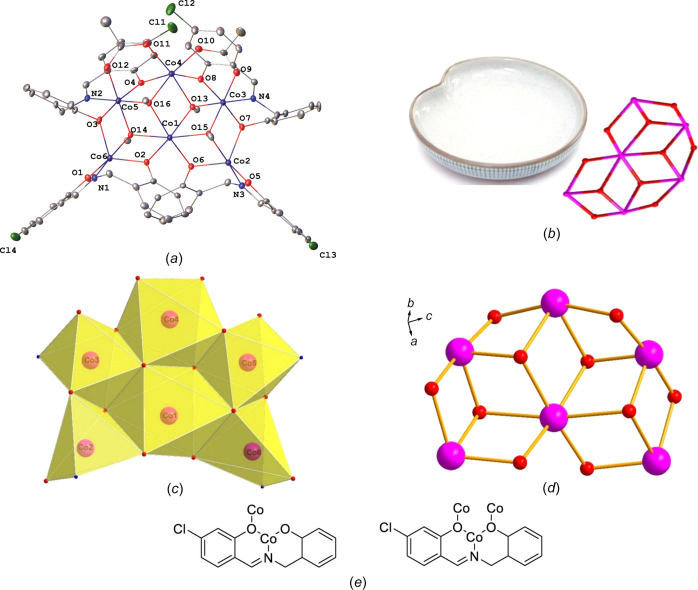
(*a*) The mol­ecular structure of **1**, (*b*) the defect disk-shaped topology, (*c*) the coordination polyhedra of the Co atoms, (*d*) the metal framework, with the H atoms omitted for clarity, and (*e*) the coordination modes of the H_2_ *L* ligand.

Single crystals of **1** were obtained from MeOH under solvothermal conditions. Complex **1** crystallized in the ortho­rhom­bic space group *P*2_1_2_1_2_1_. The structure is shown in Fig. 1 ▸. The structure analysis shows that com­plex **1** is com­posed of six cobalt ions, four 2-[(4-chloro-2-oxido­benzyl­idene­amino)­meth­yl]phenolate (*L* ^2−^) ligands, two acetate ligands and four methanol-solvent-derived MeO^−^ ligands. There exists an approximate *C*_2_ symmetry in the mol­ecule. The imine N atom and both phenolate O-atom donors of each *L* ^2−^ ligand coordinate each cobalt centre. Bond valence calculations (Brese & O’Keeffe, 1991 ▸; Brown & Altermatt, 1985 ▸) gave valence parameters of 1.90, 2.32, 3.60, 2.12, 3.64 and 2.31 for the Co1–Co6 ions, respectively, indicating that the Co3 and Co5 ions are in 3+ valence states, and that the Co1, Co2, Co4 and Co6 ions are in 2+ oxidation states. The formation of four fused defect cubes confirms the involvement of four methanol-solvent-derived μ_3_-O^−^ groups, giving the Co_6_O_10_ structure. Thus, the mol­ecular structure of **1** displays a defect disk-shaped topology [Fig. 1 ▸(*b*)]. Of the six cobalt centres, the Co1, Co3, Co4 and Co5 ions are six-coordinated, and the Co2 and Co6 ions are five-coordinated. The coordination environments of the Co2 and Co6 ions, and the Co3 and Co5 ions are individually identical. The Co1 centre is present in a distorted octa­hedral O_6_ coordination environment, among which two O atoms are from two μ_2_-κ^4^ *O*:*O*,*O*′,*N L* ^2−^ ligands and four O atoms are from four μ_3_-O^−^ MeO^−^ ligands. The Co2 centre is enclosed by the N and O atoms of one μ_2_-κ^4^ *O*:*O*,*O*′,*N L* ^2−^ ligand, one O atom of a μ_3_-κ^5^ *O*:*O*,*N*,*O*′:*O*′ *L* ^2−^ ligand and one O atom of one μ_3_-O^−^ MeO^−^ ligand. The six-coordinate NO_5_ environment around the Co3 ion is accom­plished by two μ_3_-O^−^ MeO^−^ groups, one O atom from one acetate bridge and the N and O atoms of one μ_3_-κ^5^ *O*:*O*,*N*,*O*′:*O*′ *L* ^2−^ ligand. The six O-donor atoms around the Co6 centre originate from bridging acetate ligands, two μ_3_-O^−^ MeO^−^ groups and two μ_3_-κ^5^ *O*:*O*,*N*,*O*′:*O*′ *L* ^2−^ ligands. The H_2_ *L* ligand exhibits two types of coordination mode.

The geometries of the five-coordinated Co2 and Co6 atoms were analyzed with the program *SHAPE* (Version 2.0; Pinsky & Avnir, 1998 ▸). The calculated values revealed trigonal bipyramid (*D*_3*h*_) geometry for both atoms, with a minimum CShM (contunuous shape measure) value of 1.065 for Co2 and 1.172 for Co6.

Complex **1** joins a small family of Co_6_ clusters. Hexa­nuclear cobalt com­plexes mainly exhibit wheel, cage and ring topologies (Shi *et al.*, 2021 ▸; Zou *et al.*, 2014 ▸; Wang *et al.*, 2013 ▸; Guo *et al.*, 2013 ▸; Lazzarini *et al.*, 2012 ▸; Chen *et al.*, 2010 ▸; Malassa *et al.*, 2010 ▸; Tudor *et al.*, 2010 ▸; Colacio *et al.*, 2009 ▸; Jones *et al.*, 2009 ▸; Shiga & Oshio, 2007 ▸; Alley *et al.*, 2006 ▸; Murrie *et al.*, 2003 ▸; Kumagai *et al.*, 2003 ▸; Gutschke *et al.*, 1999 ▸). Complex **1** is a rare example that displays a defect disk-shaped structure.

### Magnetic properties of 1

**Figure 2:**
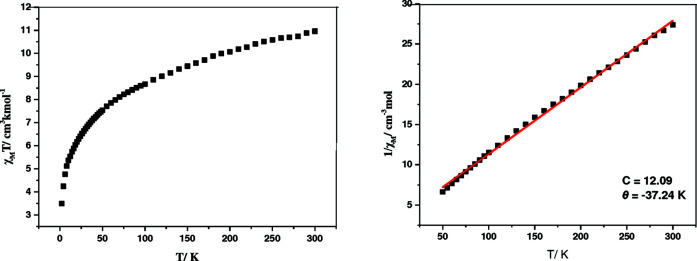
Temperature dependence of magnetic susceptibilities in the forms of (*a*) χ_M_ *T versus T* and (*b*) 1/χ_M_ *versus T* for **1** at 1 kOe. The red solid line corresponds to the best fit of the magnetic data.

Magnetic susceptibility data as a function of temperature for com­plex **1** are shown in Fig. 2 ▸. The room temperature χ_M_ *T* value is 10.96 cm^3^ mol^−1^ K, which is greater than the value of 7.50 cm^3^ mol^−1^ K for four uncoupled *S* = 3/2 Co^II^ centres, possibly owing to the orbital contributions of the metal ions (Cao *et al.*, 2013 ▸). Upon lowering the temperature, the χ_M_ *T* value drops slightly to a minimum value of 3.49 cm^3^ mol^−1^ K at 2 K, which suggests possible anti­ferromagnetic couplings between the unpaired spins. The data of 1/*χ_M_* in the temperature range 2–300 K were fitted by the Curie–Weiss Law of 1/χ_M_ = (*T* − θ)/*C*. The Curie constant *C* = 12.09 cm^3^ mol^−1^ K and the Weiss constant θ = −37.24 K were obtained. The negative θ value proves the anti­ferromagnetic inter­actions.

The magnetic dynamic behaviour of **1** was also explored. The ac magnetic susceptibilities for **1** at 1000 Hz under a zero-dc field in the temperature range 2–25 K were shown in Fig. S2 (see supporting information). The χ′′ susceptibilities at 1000 Hz did not increase upon lowering the temperature and no peaks were determined. These phenomena revealed that com­plex **1** is not a single-mol­ecule magnet.

## Conclusion

A hexa­nuclear cobalt com­plex of com­position [Co_2_^III^Co_4_^II^(*L*)_4_(CH_3_COO)_2_(MeO)_4_] (**1**), based on the hydroxy-con­taining Schiff base ligand 2-[(4-chloro-2-hy­droxy­benzyl­idene­amino)­meth­yl]phenol (H_2_ *L*) was prepared and char­acterized. Complex **1** exhibits a defect disk-shaped top­ol­ogy. Four cobalt ions are six-coordinated and two cobalt ions are five-coordinated. An investigation of the magnetic properties revealed that there exist anti­ferromagnetic inter­actions between the Co^II^ ions.

## Supplementary Material

Crystal structure: contains datablock(s) I, global. DOI: 10.1107/S2053229622005885/wv3009sup1.cif

Structure factors: contains datablock(s) I. DOI: 10.1107/S2053229622005885/wv3009Isup2.hkl

IR spectrum, magnetic susceptibility figure, geometry details and table of CShM values. DOI: 10.1107/S2053229622005885/wv3009sup3.pdf

CCDC reference: 1991979

## Figures and Tables

**Table 1:** Experimental details

Crystal data	
Chemical formula	[Co_6_(C_14_H_10_ClNO_2_)_4_(C_2_H_3_O_2_)_2_(CH_3_O)_4_]
*M*_r_	1634.52
Crystal system, space group	Orthorhombic, *P*2_1_2_1_2_1_
Temperature (K)	120
*a*, *b*, *c* (Å)	15.4873 (10), 16.2116 (11), 28.1099 (19)
*V* (Å^3^)	7057.7 (8)
*Z*	4
Radiation type	Mo *K*α
μ (mm^−1^)	1.60
Crystal size (mm)	0.4 × 0.2 × 0.2

Data collection	
Diffractometer	Bruker SMART APEXII
Absorption correction	Multi-scan (*SADABS*; Bruker, 2016 ▸)
*T*_min_, *T*_max_	0.612, 0.746
No. of measured, independent and observed [*I* > 2σ(*I*)] reflections	78040, 16170, 11376
*R*_int_	0.094
(sin θ/λ)_max_ (Å^−1^)	0.650

Refinement	
*R*[*F* ^2^ > 2σ(*F* ^2^)], *wR*(*F* ^2^), *S*	0.057, 0.174, 1.04
No. of reflections	16170
No. of parameters	853
No. of restraints	12
H-atom treatment	H-atom parameters constrained
Δρ_max_, Δρ_min_ (e Å^−3^)	0.65, −0.78
Absolute structure	Flack *x* determined using 4303 quotients [(*I* ^+^) − (*I* ^−^)]/[(*I* ^+^) + (*I* ^−^)] (Parsons *et al.*, 2013 ▸)
Absolute structure parameter	0.011 (8)

Computer programs: *SAINT* (Bruker, 2016 ▸), *APEX2* (Bruker, 2016 ▸), *olex2.solve* (Bourhis *et al.*, 2015 ▸), *SHELXL* (Sheldrick, 2015 ▸), *OLEX2* (Dolomanov *et al.*, 2009 ▸) and *PLATON* (Spek, 2020 ▸).
