# Supplementary material for: A mixed-valence [CoII 4CoIII 2] cluster with defect disk-shaped topology
Source: Acta Crystallogr C Struct Chem. 2022 Aug 26;78(Pt 9):488–92. doi: 10.1107/S2053229622005885 (PMC9444020; doi:10.1107/S2053229622005885)
Supplement: Supplementary file 3 [file c-78-00488-sup3.pdf]

Acta Cryst C

STRUCTURAL CHEMISTRY

Volume 78 (2022)

Supporting information for article:

A mixed-valence [$Co^{II}_4Co^{III}_2$] cluster with defect disk-shaped topology

Hua Yang, Yu-Pei Fu, Yuan Huang, Xiao-Li Chen, Yu-Ze Wu and Hua-Li Cui

S1. IR spectrum of 1

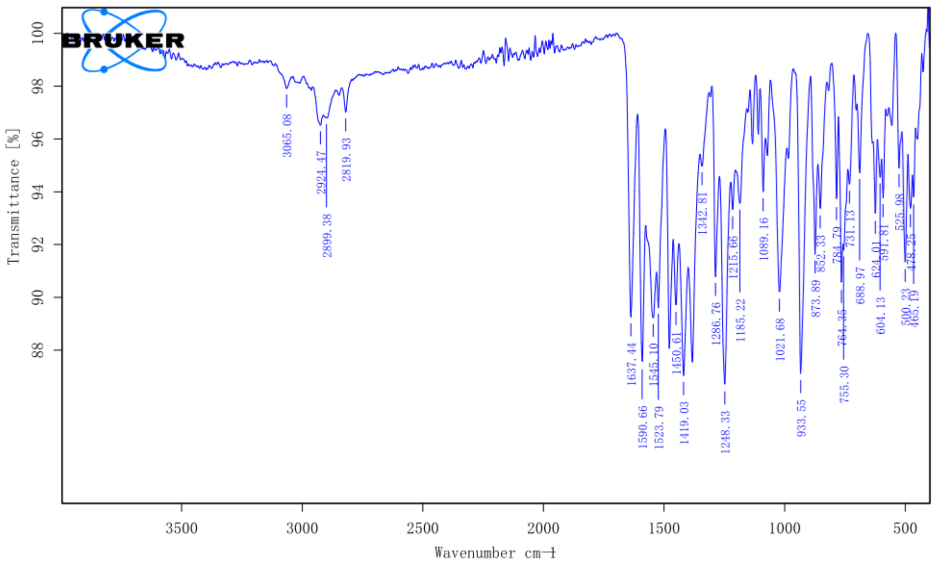

Figure S1

IR spectrum of **1**.

S2. Ac magnetic susceptibility of 1

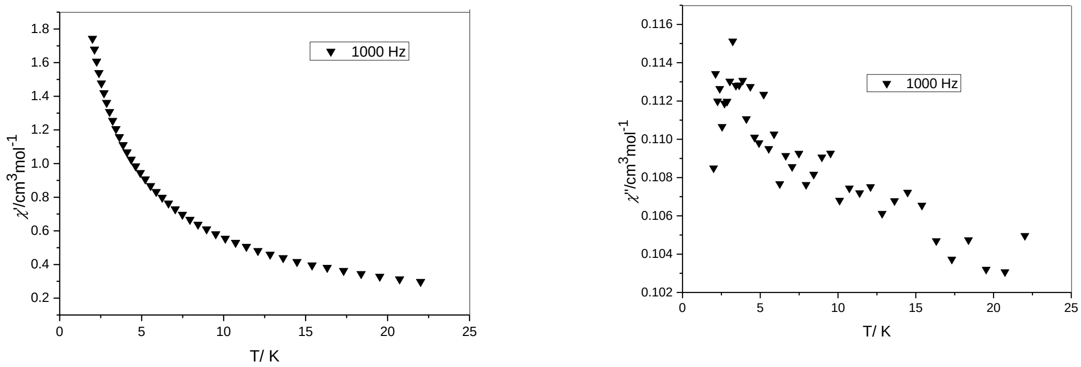

Figure S2

Temperature dependence of the in-phase (χ') (a) and out-of-phase (χ'') (b) susceptibilities for 1 in the range of 2 to 25 K. The susceptibilities at 1000 Hz frequency under zero dc field.

Table S1

*Bond distance and angles of **1***

Co1-O2	2.036(6)	Co4-O4	2.111(6)
Co1-O6	2.031(6)	Co4-O8	2.097(6)
Co1-O13	2.132(6)	Co4-O10	2.057(7)

Co1-O14	2.222(5)	Co4-O11	2.039(7)
Co1-O15	2.201(6)	Co4-O13	2.070(6)
Co1-O16	2.123(6)	Co4-O16	2.083(6)
Co2-O5	1.945(7)	Co5-O3	1.874(7)
Co2-O6	1.969(6)	Co5-O4	1.897(6)
Co2-O7	1.991(6)	Co5-O12	1.902(7)
Co2-O15	2.191(6)	Co5-O14	1.909(6)
Co2-N3	2.021(8)	Co5-O16	1.921(6)
Co3-O7	1.890(7)	Co5-N2	1.924(8)
Co3-O8	1.898(6)	Co6-O1	1.937(6)
Co3-O9	1.910(7)	Co6-O2	1.989(6)
Co3-O13	1.925(6)	Co6-O3	2.000(7)
Co3-O15	1.907(6)	Co6-O14	2.156(6)
Co3-N4	1.923(8)	Co6-N1	2.012(8)
O2-Co1-O13	170.5(2)	O8-Co4-O4	160.1(2)
O2-Co1-O14	79.2(2)	O10-Co4-O4	107.4(3)
O2-Co1-O15	106.6(2)	O10-Co4-O8	86.6(3)
O2-Co1-O16	91.9(2)	O10-Co4-O13	89.9(3)
O6-Co1-O2	97.8(3)	O10-Co4-O16	170.9(3)
O6-Co1-O13	90.8(2)	O11-Co4-O4	85.6(3)
O6-Co1-O14	106.3(2)	O11-Co4-O8	107.3(3)
O6-Co1-O15	79.6(2)	O11-Co4-O10	96.3(3)
O6-Co1-O16	169.5(3)	O11-Co4-O13	172.5(3)
O13-Co1-O14	102.1(2)	O11-Co4-O16	91.9(3)
O13-Co1-O15	71.1(2)	O13-Co4-O4	88.5(2)
O15-Co1-O14	171.3(2)	O13-Co4-O8	77.2(2)
O16-Co1-O13	79.8(2)	O13-Co4-O16	82.2(2)
O16-Co1-O14	71.5(2)	O16-Co4-O4	77.0(2)
O16-Co1-O15	101.4(2)	O16-Co4-O8	87.2(3)

O5-Co2-O6	131.1(3)	O3-Co5-O4	173.4(3)
O5-Co2-O7	119.4(3)	O3-Co5-O12	92.8(3)
O5-Co2-O15	98.7(3)	O3-Co5-O14	85.2(3)
O5-Co2-N3	92.7(3)	O3-Co5-O16	87.9(3)
O6-Co2-O7	108.0(3)	O3-Co5-N2	91.6(3)
O6-Co2-O15	81.2(2)	O4-Co5-O12	90.5(3)
O6-Co2-N3	90.6(3)	O4-Co5-O14	91.1(3)
O7-Co2-O15	75.5(2)	O4-Co5-O16	86.3(3)
O7-Co2-N3	99.8(3)	O4-Co5-N2	94.2(3)
N3-Co2-O15	168.6(3)	O12-Co5-O14	176.1(3)
O7-Co3-O8	173.8(3)	O12-Co5-O16	93.4(3)
O7-Co3-O9	91.8(3)	O12-Co5-N2	86.2(3)
O7-Co3-O13	88.2(3)	O14-Co5-O16	83.1(2)
O7-Co3-O15	84.9(3)	O14-Co5-N2	97.2(3)
O7-Co3-N4	91.9(3)	O16-Co5-N2	179.4(3)
O8-Co3-O9	90.1(3)	O1-Co6-O2	134.7(3)
O8-Co3-O13	85.7(3)	O1-Co6-O3	118.0(3)
O8-Co3-O15	92.7(3)	O1-Co6-O14	95.6(2)
O8-Co3-N4	94.0(3)	O1-Co6-N1	93.1(3)
O9-Co3-O13	93.4(3)	O2-Co6-O3	105.3(3)
O9-Co3-O15	88.0(3)	O2-Co6-O14	81.9(2)
O9-Co3-N4	174.6(3)	O2-Co6-N1	90.6(3)
O15-Co3-O13	82.2(2)	O3-Co6-O14	76.0(2)
O15-Co3-N4	96.4(3)	O3-Co6-N1	101.5(3)
N4-Co3-O13	178.6(3)	N1-Co6-O14	171.1(3)

Table S2

Fractional Atomic Coordinates (×10^4) and Equivalent Isotropic Displacement Parameters (Å^2×10^3) for ***1****. U_{eq} is defined as 1/3 of the trace of the orthogonalised U_{IJ} tensor.*

Atom	*x*	*y*	*z*	U(eq)

Co1	7653.2(7)	4803.0(7)	6304.2(4)	27.2(3)
Co2	7545.7(8)	3764.2(7)	5344.6(4)	31.0(3)
Co3	6471.1(8)	5334.0(8)	5424.9(4)	30.8(3)
Co4	6097.5(9)	6094.7(8)	6367.1(4)	33.9(3)
Co5	7107.5(8)	5756.9(8)	7248.0(4)	30.9(3)
Co6	8839.9(8)	4893.5(8)	7229.4(4)	31.1(3)
Cl1	2796.8(19)	4953(3)	7041.2(13)	88.0(13)
Cl2	6728(4)	9485.7(19)	5857.6(13)	94.9(15)
Cl3	10115(2)	2814(2)	3423.1(10)	61.8(8)
Cl4	10081(2)	2435(2)	9110.2(11)	65.7(9)
O1	8938(4)	4073(4)	7727(2)	38.0(15)
O2	8905(4)	4868(4)	6523(2)	31.2(13)
O3	8270(4)	5965(4)	7389(2)	35.9(15)
O4	5975(4)	5510(4)	7035(2)	32.8(14)
O5	8388(5)	3739(4)	4831(2)	40.9(16)
O6	7686(4)	3639(4)	6037(2)	32.6(14)
O7	6375(4)	4225(4)	5223(2)	35.2(15)
O8	6589(4)	6401(4)	5695(2)	34.4(14)
O9	5259(4)	5511(4)	5349(2)	42.2(16)
O10	4897(5)	6094(5)	6053(2)	45.3(17)
O11	5940(6)	7169(4)	6734(2)	52(2)
O12	6775(5)	6864(4)	7377(2)	43.2(17)
O13	6353(4)	4952(4)	6070(2)	29.8(13)
O14	7492(4)	4674(4)	7086.3(19)	28.9(13)
O15	7654(4)	5070(4)	5537(2)	28.8(13)
O16	7373(4)	5983(4)	6593(2)	30.1(13)
N1	10097(5)	5200(5)	7269(3)	34.2(17)
N2	6850(5)	5540(5)	7906(3)	36.4(19)
N3	7286(5)	2548(5)	5280(3)	35.2(18)
N4	6619(5)	5716(5)	4782(3)	36.4(18)
C1	4468(6)	5295(7)	7069(4)	45(3)
C2	3754(7)	5145(9)	7339(4)	60(3)
C3	3781(7)	5108(8)	7839(4)	53(3)
C4	4559(7)	5190(7)	8052(4)	49(3)
C5	5320(6)	5333(6)	7801(4)	39(2)
C6	5283(6)	5380(6)	7300(4)	37(2)
C7	6097(7)	5405(6)	8072(3)	38(2)

C8	7583(7)	5597(6)	8253(3)	36(2)
C9	8089(7)	6371(6)	8189(3)	38(2)
C10	8217(8)	6933(7)	8549(4)	48(3)
C11	8722(8)	7628(7)	8482(4)	52(3)
C12	9110(8)	7766(7)	8028(4)	52(3)
C13	8968(8)	7219(6)	7656(4)	45(2)
C14	8449(7)	6517(6)	7750(3)	39(2)
C15	9556(7)	3343(7)	8366(4)	44(2)
C16	9615(6)	3927(6)	8002(3)	35(2)
C17	10425(6)	4339(6)	7952(3)	35(2)
C18	10999(7)	3583(8)	8636(4)	51(3)
C19	11099(7)	4154(7)	8276(4)	47(3)
C20	10212(7)	3171(7)	8668(4)	44(2)
C21	10635(6)	4918(6)	7579(3)	35(2)
C22	10409(7)	5708(7)	6875(3)	40(2)
C23	10413(6)	5210(7)	6432(3)	39(2)
C24	11158(7)	5121(6)	6156(4)	42(2)
C25	11186(7)	4631(7)	5759(3)	44(2)
C26	10474(7)	4197(7)	5621(4)	43(2)
C27	9702(6)	4266(6)	5882(3)	36(2)
C28	9662(6)	4782(6)	6278(3)	34(2)
C29	8365(6)	2853(6)	6654(3)	37(2)
C30	8427(7)	2144(6)	6922(4)	43(2)
C31	7947(7)	1476(7)	6794(4)	48(3)
C32	7422(7)	1498(6)	6391(4)	45(2)
C33	7353(6)	2198(6)	6117(3)	33(2)
C34	7802(6)	2900(5)	6267(3)	32(2)
C35	6792(6)	2187(6)	5677(3)	37(2)
C36	7550(6)	2086(6)	4935(3)	36(2)
C37	8119(6)	2338(6)	4560(3)	36(2)
C38	8302(7)	1754(7)	4205(3)	43(2)
C39	8873(7)	1895(7)	3846(3)	47(3)
C40	9318(7)	2643(8)	3847(3)	47(3)
C41	9149(7)	3239(7)	4177(4)	42(2)
C42	8532(6)	3123(6)	4540(3)	35(2)
C43	5196(6)	3442(7)	4902(4)	42(2)
C44	5833(6)	4050(6)	4854(3)	35(2)

C45	4645(7)	3265(8)	4515(4)	55(3)
C46	4723(7)	3731(9)	4106(4)	56(3)
C47	5346(8)	4342(8)	4064(4)	53(3)
C48	5921(7)	4488(6)	4441(3)	40(2)
C49	6648(7)	5090(6)	4405(3)	41(2)
C50	6674(6)	6489(6)	4657(3)	36(2)
C51	6675(6)	7168(6)	4969(3)	36(2)
C52	6742(7)	7961(6)	4756(4)	45(3)
C53	6754(8)	8669(7)	5016(4)	54(3)
C54	6692(8)	8603(7)	5515(4)	51(3)
C55	6638(8)	7861(6)	5741(4)	45(3)
C56	6622(6)	7123(6)	5466(3)	36(2)
C57	4733(7)	5833(8)	5639(4)	53(3)
C58	3814(8)	5903(13)	5464(6)	96(5)
C59	6256(11)	7309(7)	7142(4)	62(4)
C60	6036(12)	8124(10)	7369(6)	94(5)
C61	5769(6)	4302(6)	6171(3)	36(2)
C62	7050(6)	3996(6)	7304(3)	36(2)
C63	7956(7)	6638(6)	6484(3)	37(2)
C64	8312(6)	5582(6)	5321(4)	40(2)

Table S3

*Anisotropic Displacement Parameters (Å^2×10^3) for **1**. The Anisotropic displacement factor exponent takes the form: $-2\pi^2[h^2a^{*2}U_{11}+2hka^*b^*U_{12}+...]$.*

**Atom**	**U_{11}**	**U_{22}**	**U_{33}**	**U_{23}**	**U_{13}**	**U_{12}**
Co1	30.6(6)	26.9(6)	24.2(6)	-2.6(5)	-1.5(5)	0.2(5)
Co2	34.6(6)	30.0(6)	28.3(6)	-3.2(5)	-2.7(5)	2.4(5)
Co3	34.9(6)	32.9(7)	24.6(6)	-0.6(5)	-3.1(5)	3.8(5)
Co4	40.6(7)	33.6(6)	27.5(6)	-0.9(5)	0.1(5)	8.6(6)
Co5	38.4(7)	30.4(6)	24.0(6)	-2.8(5)	1.7(5)	1.0(5)
Co6	31.5(6)	35.8(6)	26.1(6)	-2.7(5)	-1.5(5)	-2.3(5)
Cl1	34.2(14)	163(4)	67(2)	9(2)	3.6(13)	13(2)
Cl2	189(5)	36.1(16)	60(2)	-4.1(14)	5(2)	-7(2)
Cl3	63.2(18)	78(2)	43.6(15)	15.6(15)	17.8(14)	16.8(16)
Cl4	63.4(19)	77(2)	56.2(18)	29.2(16)	14.0(15)	19.3(16)
O1	32(3)	45(4)	38(4)	1(3)	-5(3)	-4(3)
O2	29(3)	41(3)	24(3)	-1(3)	0(2)	-1(3)

O3	48(4)	37(4)	23(3)	-7(3)	4(3)	-8(3)
O4	33(3)	36(3)	29(3)	2(3)	4(3)	6(3)
O5	51(4)	37(4)	36(3)	-4(3)	-1(3)	3(3)
O6	40(4)	29(3)	28(3)	-1(3)	-4(3)	2(3)
O7	37(3)	42(4)	28(3)	-3(3)	-8(3)	-1(3)
O8	52(4)	28(3)	24(3)	3(3)	-1(3)	3(3)
O9	40(4)	52(4)	34(4)	3(3)	-2(3)	5(3)
O10	45(4)	52(4)	40(4)	4(3)	1(3)	13(3)
O11	85(6)	37(4)	34(4)	-1(3)	3(4)	21(4)
O12	67(5)	33(4)	29(3)	-1(3)	5(3)	1(3)
O13	35(3)	28(3)	26(3)	1(2)	-1(2)	0(3)
O14	34(3)	31(3)	22(3)	-1(2)	2(2)	-3(3)
O15	32(3)	29(3)	26(3)	-4(2)	-2(2)	-1(3)
O16	36(3)	29(3)	26(3)	-3(2)	-1(3)	-3(3)
N1	39(4)	34(4)	30(4)	0(3)	-6(3)	-7(4)
N2	51(5)	32(4)	26(4)	-4(3)	0(3)	2(4)
N3	43(4)	31(4)	31(4)	-3(3)	-2(3)	2(3)
N4	34(4)	46(5)	29(4)	-5(4)	-5(3)	4(4)
C1	37(5)	66(7)	32(5)	-2(5)	7(4)	14(5)
C2	35(6)	83(9)	61(7)	-4(7)	10(5)	9(6)
C3	38(6)	70(8)	51(6)	-12(6)	16(5)	1(5)
C4	52(6)	53(7)	40(6)	-9(5)	14(5)	8(6)
C5	40(5)	39(5)	39(5)	-2(5)	7(4)	5(4)
C6	31(5)	40(5)	40(5)	-6(4)	15(4)	3(4)
C7	46(6)	37(5)	31(5)	0(4)	9(4)	-2(5)
C8	43(5)	41(5)	24(4)	-2(4)	0(4)	-4(4)
C9	52(6)	44(6)	18(4)	-2(4)	-5(4)	1(5)
C10	62(7)	53(6)	30(5)	-14(5)	-6(5)	6(6)
C11	58(7)	46(6)	51(6)	-21(5)	-11(6)	4(5)
C12	63(7)	38(6)	55(7)	-8(5)	-9(6)	-6(5)
C13	60(7)	35(5)	40(6)	-7(4)	0(5)	-11(5)
C14	51(6)	35(5)	31(5)	-10(4)	-4(5)	-1(4)
C15	43(6)	49(6)	39(6)	7(5)	8(5)	7(5)
C16	36(5)	42(5)	27(4)	-5(4)	3(4)	7(4)
C17	39(5)	37(5)	28(4)	-1(4)	-1(4)	-2(4)
C18	45(6)	71(8)	38(6)	7(6)	-5(5)	12(6)
C19	40(6)	55(7)	47(6)	-1(5)	-10(5)	4(5)

C20	47(6)	56(6)	29(5)	5(5)	1(4)	18(5)
C21	30(5)	39(5)	38(5)	1(4)	0(4)	-3(4)
C22	44(6)	42(6)	34(5)	7(4)	-3(4)	-5(5)
C23	34(5)	45(6)	38(5)	10(5)	-4(4)	-7(4)
C24	41(5)	39(5)	46(6)	14(5)	8(4)	0(5)
C25	38(5)	58(7)	35(5)	15(5)	12(4)	3(5)
C26	44(6)	47(6)	37(5)	7(5)	10(4)	19(5)
C27	37(5)	39(5)	32(5)	2(4)	-4(4)	11(4)
C28	37(5)	37(5)	26(4)	1(4)	5(4)	8(4)
C29	41(5)	36(5)	33(5)	-2(4)	1(4)	2(4)
C30	50(6)	42(6)	37(5)	9(5)	1(5)	10(5)
C31	55(7)	38(6)	50(6)	3(5)	-6(5)	-1(5)
C32	61(7)	26(4)	48(6)	3(4)	0(5)	-7(5)
C33	44(5)	29(4)	27(4)	4(4)	-1(4)	5(4)
C34	37(5)	23(4)	35(5)	-4(4)	-3(4)	6(4)
C35	42(5)	35(5)	34(5)	-6(4)	5(4)	-7(4)
C36	40(5)	32(5)	38(5)	-11(4)	1(4)	-4(4)
C37	40(5)	42(5)	24(4)	-3(4)	-7(4)	9(4)
C38	45(6)	51(6)	33(5)	-6(5)	4(4)	-1(5)
C39	55(7)	56(7)	29(5)	-6(5)	-4(5)	13(6)
C40	49(6)	67(8)	24(5)	11(5)	2(4)	19(6)
C41	44(6)	40(6)	41(6)	9(5)	7(5)	2(5)
C42	36(5)	40(5)	31(5)	0(4)	2(4)	11(4)
C43	37(5)	49(6)	41(6)	-4(5)	-10(4)	-9(5)
C44	31(5)	41(5)	31(5)	-10(4)	-5(4)	3(4)
C45	38(6)	74(8)	55(7)	-21(6)	-7(5)	-7(6)
C46	46(6)	94(10)	29(5)	-16(6)	-14(5)	-16(7)
C47	59(7)	72(8)	28(5)	-10(5)	-10(5)	15(6)
C48	48(6)	47(6)	24(4)	-7(4)	-1(4)	4(5)
C49	63(6)	38(5)	21(4)	0(4)	-2(4)	6(5)
C50	33(5)	41(5)	34(5)	4(4)	1(4)	3(4)
C51	37(5)	44(6)	27(5)	6(4)	-2(4)	4(4)
C52	56(6)	42(6)	37(5)	12(5)	-11(5)	-10(5)
C53	75(8)	36(6)	49(7)	11(5)	-3(6)	-10(6)
C54	72(8)	41(6)	41(6)	4(5)	-5(5)	-3(6)
C55	71(7)	30(5)	35(5)	4(4)	-9(5)	0(5)
C56	40(5)	33(5)	34(5)	10(4)	-2(4)	13(4)

C57	47(6)	71(8)	40(6)	3(6)	0(5)	28(6)
C58	39(7)	162(15)	87(10)	-24(11)	-15(7)	37(9)
C59	121(12)	38(6)	27(5)	-2(4)	5(6)	21(7)
C60	98(5)	91(5)	92(5)	-1(3)	-1(3)	4(3)
C61	32(5)	40(5)	38(5)	1(4)	-2(4)	-7(4)
C62	33(5)	34(5)	41(5)	3(4)	5(4)	-4(4)
C63	50(6)	26(5)	35(5)	1(4)	-3(4)	-5(4)
C64	43(5)	40(5)	38(5)	11(4)	-10(4)	-12(4)

Table S4

CshM values of Co2 and Co6 atoms calculated by SHAPE 2.0 Program

	PP-5	vOC-5	TBPY-5	SPY-5	JTBPY-5
Co2	31.492	5.930	1.065	5.198	2.813
Co6	31.623	4.889	1.172	4.211	3.051
